# An Inverse Approach of Damage Identification Using Lamb Wave Tomography

**DOI:** 10.3390/s19092180

**Published:** 2019-05-10

**Authors:** Yaolu Liu, Shijie Zhou, Huiming Ning, Cheng Yan, Ning Hu

**Affiliations:** 1 College of Aerospace Engineering, Chongqing University, Chongqing 400044, China; 20132283@cqu.edu.cn; 2 Chongqing Key Laboratory of Heterogeneous Material Mechanics, Chongqing University, Chongqing 400044, China; 3 School of Chemistry, Physics and Mechanical Engineering, Science and Engineering Faculty, Queensland University of Technology (QUT), Brisbane, QLD 4001, Australia; c2.yan@qut.edu.au; 4 School of Mechanical Engineering, Hebei University of Technology, Tianjin 300401, China; 5 State Key Laboratory of Coal Mine Disaster Dynamics and Control, Chongqing University, Chongqing 400044, China

**Keywords:** lamb wave tomography, inverse algorithm, damage, image, wave attenuation, rapid inspection technique

## Abstract

A pulse laser combined LWT technique with a two-stage reconstruction algorithm was proposed to realize rapid damage location, or even the evaluation of damage size for plate-like structures. Since the amplitude of Lamb waves in propagation is highly sensitive to damage, including inside damage, the change of the attenuation coefficient of Lamb waves in the inspection region was used as a damage index to reconstruct damage images. In stage one, the rough area of the damage was identified by a comparison of the amplitude of the testing signal data and reference data (undamaged state). In stage two, the damage image was reconstructed using an inverse approach based on the least-square method. In order to verify the effectiveness of the proposed rapid approach, experiments on an aluminum plate with a non-penetrating notch and a carbon fiber-reinforced plastic laminated plate with internal delamination induced by a low-velocity impact were carried out. The results show that the notch can be detected with accurate location, and the delamination image can be reconstructed successfully.

## 1. Introduction

To prevent fatigue, corrosion and aging failure in engineering structures, it is essential to develop various rapid non-destructive testing (NDT) technologies to estimate structural safety and integrity. Since plate or shell structures are widely used in aerospace, energy and chemical engineering fields, and they commonly have a very large size, it calls for a kind of NDT technique especially developed for those structures. Lamb waves are a special kind of ultrasonic guide wave which arises from a coupling between shear and longitudinal waves reflected at the top and bottom of a plate-like structure. Lamb waves can travel relatively long distances in structures and have high sensitivity to damage even inside structures; therefore, Lamb wave-based NDT techniques [1,2,3,4] are commonly employed to inspect plate or shell structures in comparison to other damage detection techniques.

Computed tomography (CT) [5,6] is a well-known damage image technique in NDT that has been successfully applied to medical diagnosis, geophysical field, instrumentation, and industrial process monitoring. For plate or shell structures, an ultrasonic Lamb waves-based tomographic reconstruction technique, or simply named Lamb wave tomography (LWT) [7,8], has been developed. This technique is orientated to various possible engineering applications of NDT, e.g., the detection of material loss; delamination in composites; and debonding of connection components or joints, cracks, borehole, etc. Different from the conventional CT technique that is to reconstruct cross-section pictures of a structure, the LWT technique is to reconstruct in-plane surface images of the plate or shell structures by manipulating numerous different rays of Lamb waves passing through the plane of interest. Hutchins et al. [9] firstly put forward the LWT technique to image defects in thin aluminum plates using a pulsed laser source and an electromagnetic acoustic transducer (EMAT) detector. Then Jansen et al. [10] reconstructed the damage region in two polymer composite plates using a Lamb wave tomography technique. Later, a number of studies in time-of-fight LWT technique were carried out by the research team led by Hinders [11,12,13,14,15]. Recent studies have dedicated their efforts to improve the resolution of LWT and promote the realization of engineering applications. Huang et al. [16] proposed a multi-mode electromagnetic ultrasonic LWT method for variable-depth defects in metal plates, and the imaging results show that the computed thickness distribution is more accurate than the ones of the traditional signal-mode LWT method. Chen et al. [17] developed a time-of-flight revising LWT method for composite panels to improve the image quality. The time-of-flight data of flawed plates are revised according to baseline data obtained from an unflawed plate to reduce the effects of anisotropy on image reconstruction. Seher et al. [18] performed field trials for oil pipelines with restricted access of the inspection area using an EMAT-based guided wave tomography system. They employed a kind of hybrid algorithm that combines a bent-ray tomography algorithm with a diffraction tomography algorithm to compute the thickness map for pipes. Zhao et al. [19] put forward probabilistic diagnostic algorithm-based damage detection using the improved weight function for non-uniform-section plates, in which the reference data (undamaged state) were used to assess the probability of the damage.

It has to be mentioned that another approach to improve the image quality and resolution of LWT is by incorporating the diffraction effect in the reconstruction. Malyarenko et al. [20] firstly proposed the Lamb wave diffraction tomography technique. Belanger et al. [21] developed a Low-frequency-guided wave diffraction tomography within the born approximation. A circular transducer network with 64 transducers is used to estimate the thickness reductions of plates. The results showed that the location, size and depth of the damage can be accurately evaluated. Ng et al. [22] proposed a two-stage imaging approach based on the cross-correlation analysis and Lamb wave diffraction tomography for quantitative imaging of damages in metallic plates. They successfully reduced the transducer number of the circular transducer network to 8 based on the proposed approach. Finite element simulation results showed that the damage could be accurately located with an inaccuracy of the order of a few millimeters of a circular inspection of 100 mm^2^ and proved a reasonable estimation of the size and depth of the damages. Chan et al. [23] developed a near-field diffraction tomography using numerical Green’s functions for quantitative characterization of laminar damage in plate-like structures. Results showed that the proposed approach was capable of determining the damage geometry, including multi-site damage. However, the approach required the number of transducers satisfying the Nyquist sampling criterion for the size of the imaging domain.

**Figure 1:**
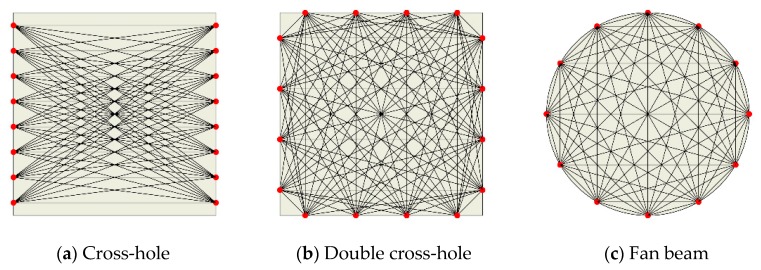
Transducer array of conventional LWTs.

Similar to the CT technique, the changes of time-of-flight [11,12,13,14,24,25,26] and wave amplitude [27,28,29] are both commonly used in LWT as signal parameters in image reconstruction algorithms, which are related to wave velocity and wave attenuation respectively. For the early ultrasonic reconstruction algorithms, the classical Fourier inversion technique [30] and filtered back-projection technique [9,10,31,32] have been used to reconstruct the image by referring to the X-ray CT technique. However, all of those classical techniques have certain limitations, such as low capability of anti-noise interference, high requirement for precise sampling geometry, etc. Considering that the wave propagation path is a fold line due to the change of wave velocity in the damage region, the iterative reconstruction algorithms of seismic wave tomography have also been applied to ultrasonic tomography. The iterative reconstruction algorithms include an algebraic reconstruction technique [19,33], simultaneous iterative reconstruction technique [13,34,35,36] and statistical iterative method implemented by some iteration algorithms of maximum likelihood expectation [37,38]. However, the iterative reconstruction algorithm-based techniques have the disadvantage of the iterative convergence problem and low computational efficiency. In addition, the schemes for the transducer array of conventional LWTs usually employ linear array configuration such as cross-hole [11] and double cross-hole [12], and the circular array configuration with fan beam projection [12] as shown in Figure 1. Transducers on the boundary of the inspection region both transmit and receive wave signals. It can be found that those schemes need many transducers or to move the transducers frequently when only using a pair of transducers.

In this paper, a pulse laser-combined LWT technique with a two-stage reconstruction algorithm was put forward to realize quick inspections for plate-like structures. In stage one, the rough area of the damage was located by comparing the testing signal data and reference data (undamaged state). In stage two, the damage image was reconstructed using an inverse approach based on the least-square method. The experimental investigations on an aluminum plate with a non-penetrative notch and on a carbon fiber-reinforced plastic (CFRP)-laminated plate with internal delamination induced by a low-velocity impact are carried out to confirm the effectiveness of the proposed technique. This paper is organized as follows. In Section 2, the experimental scheme of this new technique is introduced. In Section 3, the reconstruction algorithm is described in detail. In Section 4, this technique is validated experimentally by detecting two different types of damage in the aluminum plate and the CFRP laminated plate. Finally, some conclusions are drawn in Section 5.

## 2. Experimental Scheme

**Figure 2:**
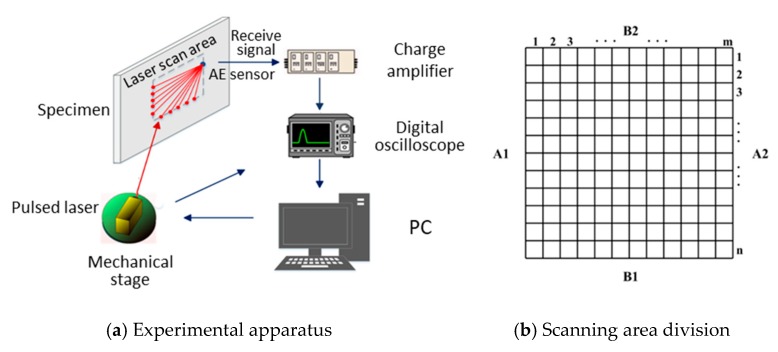
Schematic of experiments.

Figure 2a presents a schematic overview of experiments for the data acquisition. A pulse laser is used to generate Lamb waves based on a thermoelastic mechanism. The excited Lamb waves depends on the duration of the used laser pulse and the material properties. The duration of the used laser pulse is 8.5 ns (120 MHz), resulting in a wave spectrum in which the wave energy is mainly distributed from 100 kHz~300 kHz for the aluminum and CFRP specimens. Table 1 and Table 2 present the material properties of the aluminum and CFRP specimens used in the following experiments. An acoustic emission (AE) sensor attached on a particular location of a plate is employed to receive wave signals. In the experiment, a rectangular inspection region needs to be determined firstly, which is divided by grids m×n as shown in Figure 2b. The size of the inspection region depends on the attenuation of wave propagation. In general, the inspection region of aluminum specimens is larger than that of CFRP specimens since the wave attenuation is more severe in the CFRP composites. The size of the grids is determined by the wavelength of the detection waves in the experiment, and it is usually to divide into at least five grids in one wavelength to effectively describe the attenuation of the waves. For the convenience of the later description, the four edges of the rectangular inspection region are defined as A1, B1, A2, and B2 in counterclockwise direction as shown in Figure 2b.

**Figure 3:**
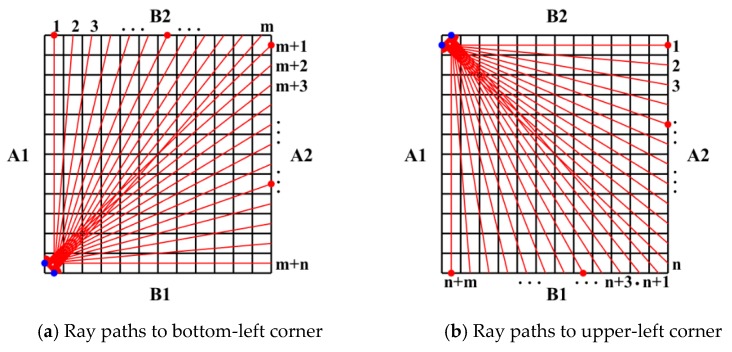
Scheme of grids division and data acquisition (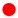: Laser position, 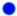: Sensor).

The experimental data-collecting scheme is shown in Figure 3. In Figure 3a, the laser irradiates step-by-step along the B2 and A2 to sweep all available positions to generate Lamb waves; meanwhile, the AE sensor is fixed in the bottom-left corner (the first grid of B1 edge and the last grid of A1 edge) for data acquisition. In Figure 3b, the ray paths start from the first grid of the A1 edge to all grids of the A2 edge, and from the first grid of the B2 edge to all grids of the B1 edge. Thus, two sets of signal data are obtained. We can see that both of the ray paths in Figure 3a,b are covering the whole inspection region, which implies the obtained experimental data can be used to evaluate the integrity of the inspection region. In addition, it should be noted that the data-collecting scheme can be realized with high efficiency because of automatic laser-scanning and infrequent position change of the AE sensor.

**Figure 4:**
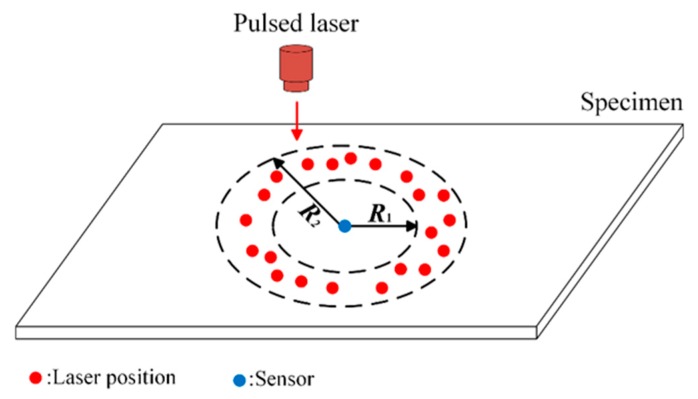
Experimental scheme of reference measurements.

Furthermore, reference measurements in an intact region need to be performed to realize damage image reconstruction. Figure 4 presents the experimental scheme for reference data acquisition. The pulse laser randomly irradiates a intact ring region in the plate, and a sensor in the center of the ring region receives wave signals propagating over different distances and along different directions. Based on those wave signals, reference data related to the ray paths in Figure 3 can be calculated by linear interpolation.

## 3. Reconstruction Algorithm

The computation of damage image is achieved in the following two steps. The first step is to roughly identify damage areas and locations by selecting the ray paths through the damaged region. We extract the wave amplitude of each ray path as the characteristic parameter for calculation. The wave amplitude is denoted by $H_{1i}$ (*i* = 1, 2, 3, …, *m + n*) for each *i*th ray path in Figure 3a. For comparison, the reference value $H_{ref-1i}$ is estimated by the reference measurements in Figure 4, which refers to the wave amplitude when the excitation wave propagates the same direction and distance as those of $H_{1i}$ in the *i*th ray path for the damaged specimen. The $H_{1i}$ would be much different to the $H_{ref-1i}$ if there is damage existing in the inspection region. For the case of the hole-shaped damage, the wave may be largely reflected back when encountering the damage, so the transmitted waves of the ray path passing through the damaged region decrease severely, and the ray path can be selected by the following equation:

(1) $$\frac{H_{1i}}{H_{ref-1i}}<\alpha_{1}$$

where $\alpha_{1}$ is a threshold smaller than 1.

However, for the case of the crack-like damage, when the direction of wave propagation is oblique or even approximatively parallel to the crack length direction, the wave may not only be weakly reflected, but also be diffracted, so the transmitted waves of the ray path passing through the damaged region may increase anomaly. Therefore, the following equation

(2) $$\frac{H_{1i}}{H_{ref-1i}}>\alpha_{2}$$

is also considered to select the ray path passing through the damaged region. $\alpha_{2}$ is a threshold larger than 1. Finally, a set of ray paths passing through the suspicious damaged region can be selected.

Similarly, the wave amplitude is denoted by $H_{2i}$ (*i* = 1, 2, 3, …, *m + n*) for each *i*th ray path in Figure 3b. $H_{ref-2i}$ is the reference value of the 2*i*th ray path. Another set of ray paths passing through the damaged region can be selected by the same procedure stated above using the following equations

(3) $$\frac{H_{2i}}{H_{ref-2i}}<\alpha_{3}\;\mathrm{and}\;\frac{H_{2i}}{H_{ref-2i}}>\alpha_{4}$$

where $\alpha_{3}$ is a threshold smaller than 1, and $\alpha_{4}$ is another one larger than 1.

**Figure 5:**
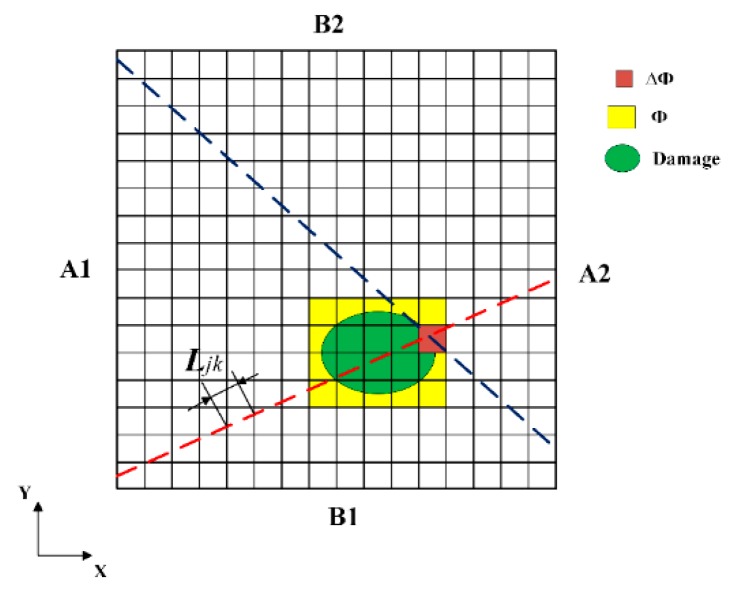
Models of estimated possible damaged area.

After selecting the two sets of ray paths passing through the damaged region, the region of ray-path intersection ΔΦ as shown in Figure 5 is considered as the initial suspected region, and the sum of ΔΦ, as marked by Φ, is the rough area of the possible damage. As a result, the possible damage regions can be localized, leading to the significantly low computational cost in the following step.

The second step is to reconstruct damaged images. For each grid in the inspection region, the attenuation coefficient of the waves is denoted by $x_{k}\;\left(k=1,\;2,\;3,\cdots ,m\times n\right)$, and *k* is the serial number of each grid. So, the amplitude attenuation of an arbitrary ray path can be linearly approximated as follows:

(4) $$D_{j}=\sum_{k=1}^{m\times n}L_{jk}x_{k}^{}$$

where *j* is an arbitrary ray path, and $D_{j}$ is a positive value, which denotes the amplitude attenuation when Lamb waves propagate from the excitation point to the sensor position along the *j*th ray path. $L_{jk}$ is the segment length of the *j*th ray path in the *k*th grid, as shown in Figure 5. We assume that the attenuation coefficient of the waves would change in the damaged region due to the effect of the damage. Therefore, the attenuation coefficient of the grid in the possible damaged region Φ determined by the first step can be expressed as follows:

(5) $$x_{k}^{D}=x_{k}^{I}+\Delta x_{k}$$

where $\Delta x_{k}$ is the attenuation coefficient change of the *k*th grid in the region Φ due to the possible damage, compared with the attenuation coefficient $x_{k}^{I}$ in intact status. $x_{k}^{D}$ is the attenuation coefficient of the *k*th grid in damage status. Therefore, for the ray path passing through the possible damage region Φ, the amplitude attenuation can be rewritten as:

(6) $$D_{j}=\sum_{k\notin \phi }L_{jk}x_{k}^{I}+\sum_{k\in \phi }L_{jk}x_{k}^{D}$$

The first term of the right side represents the sum of amplitude change due to natural attenuation when the wave passes through the grids out of the Φ region, and the second term is the sum of amplitude change caused by the attenuation in the Φ region. Then, the following equation can be obtained by substituting Equation (5) into Equation (6),

(7) $$\sum_{k\in \phi }L_{jk}\Delta x_{k}=D_{j}-\sum_{k\in \phi }L_{jk}x_{k}^{I}-\sum_{k\notin \phi }L_{jk}x_{k}^{I}=f_{j}$$

where $f_{i}$ denotes the amplitude change of the *j*th ray path caused by damage.

Considering the information in the first step, we can know that

(8) $$f_{i}=H_{j}-H_{ref-j}$$

where $H_{j}$ is the wave amplitude at the AE sensor point of the *j*th ray path and $H_{ref-j}$ is reference value of the *j*th ray path estimated in the intact region. Therefore, Equation (7) can be expressed as follows:

(9) $$\sum_{k\in \phi }L_{jk}\Delta x_{k}=H_{j}-H_{ref-j}=f_{j}$$

The vector notation of Equation (9) can be expressed as:

(10) LΔx=f

which can be easily solved by using an inverse approach based on the least-square method as follows:

(11) $$\Delta x={\left(L^{T}L\right)}^{-1}\left(L^{T}f\right)$$

The above equation yields $\Delta x_{k}$, i.e., the change of attenuation coefficient; the damage extents in various grids of the possible damaged zone can be represented by $\Delta x_{k}$ and plotted to reconstruct the damaged image in detail.

## 4. Results and Discussion

In this section, two specimens with different kinds of damage were used to experimentally validate the effectiveness of the proposed technique. The details of experiments, determination of threshold values, and those results were discussed in detail.

### 4.1. An Aluminum Plate with a Non-Penetrating Notch

**Figure 6:**
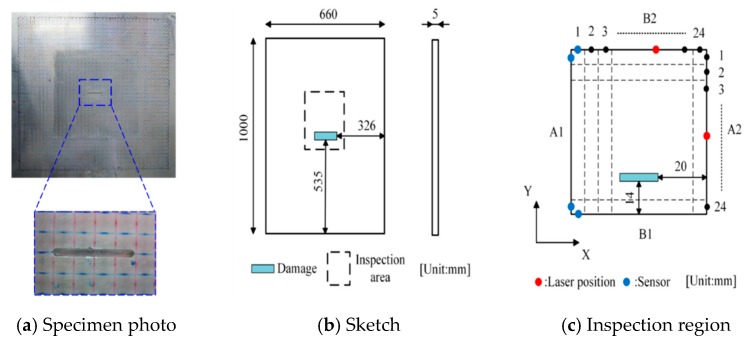
Schematic view of the aluminum plate.

**Figure 7:**
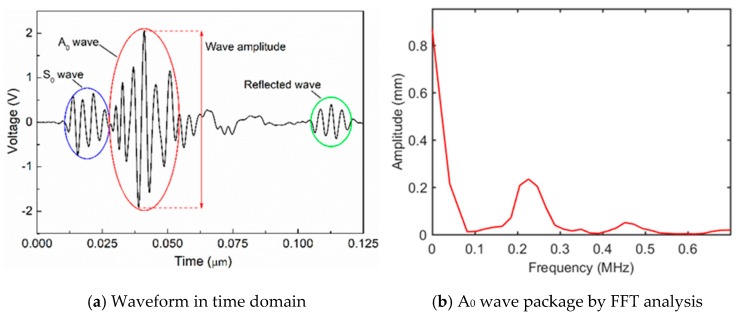
Typical wave signal received by AE sensor.

A rectangular aluminum plate (thickness: 5.0 mm) containing a non-penetrating notch (length: 20 mm, width: 2 mm and depth: 2.5 mm), as shown in Figure 6a,b, was used. By considering the wave attenuation in the aluminum plate, the inspection region was determined as 60 × 60 mm^2^ and divided into 24 × 24 grids with a side length of 2.5 mm (Figure 6c). According to the data-collecting scheme shown in Figure 3a,b (*m* = 24, *n* = 24), the pulse laser irradiated along the specified paths (B2→A2 and A2→B1), meanwhile, an AE sensor was placed in the appropriate location (the solid blue circles in Figure 3a,b) to receive wave signals. As a result, only 24 × 4 = 96 waveform data were used in this inspection. Figure 7 presents a typical wave signal received by the AE sensor. We can see from Figure 7a that the main wave mode in the waveform is the second strong wave packet, corresponding to the A_0_ mode. The frequency of the A_0_ mode wave packet is mainly in range of 200 kHz to 300 kHz as shown in Figure 7b. After extracting the amplitudes of the second wave packet in the waveforms of all ray paths, i.e., $H_{1i}$ and $H_{2i}\;\left(i=1,\;2,\;\cdots ,\;48\right)$, the reconstruction algorithm was employed to calculate the damage image.

For comparison, reference measurements used another intact aluminum plate which was performed to obtain the reference data, i.e., $H_{ref-1i}$ and $H_{ref-2i}\;\left(i=1,\;2,\cdots ,\;48\right)$ on the basis of the experimental scheme in Figure 4. The inner radius *R*_1_ and outer radius *R*_2_ of the ring were set to be 60 mm and 80 mm according to the shortest and longest wave propagation distances in the inspection region. Since the aluminum is isotropic, the reference data are only related to the wave propagation distances, which can be estimated by one-dimensional linear interpolation.

**Figure 8:**
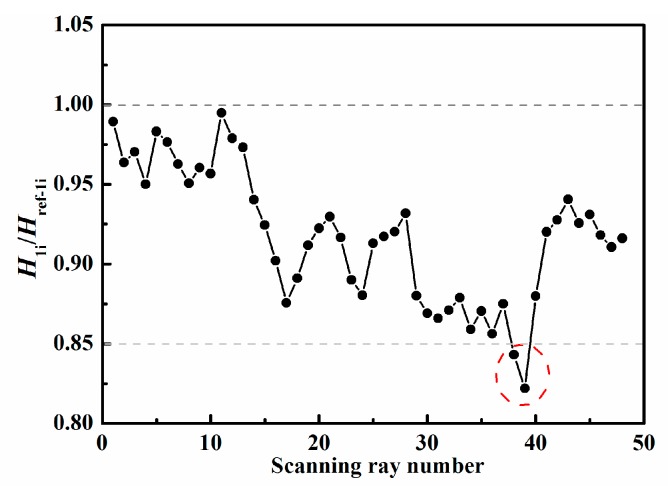
Determination of threshold values for ray paths to bottom-left corner.

**Figure 9:**
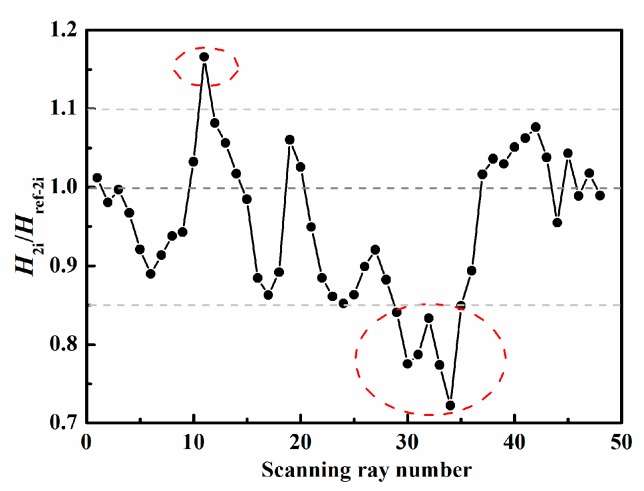
Determination of threshold values for ray paths to upper-left corner.

To roughly identify the area and location of damage, the four thresholds $\alpha_{1},\;\alpha_{2},\;\alpha_{3}$, and $\alpha_{4}$ in Equations (1)–(3) need to be determined. Figure 8 and Figure 9 present the ratios of the measured value $H_{1i}$ (or $H_{2i}$) to the reference value $H_{ref-1i}$ (or $H_{ref-2i}$) corresponding to the ray paths in Figure 3a,b, respectively. In general, for the ray path without damage, this ratio value, i.e., $H_{1i}/H_{ref-1i}$ (or $H_{2i}/H_{ref-2i}$), floating up and down by 10–20%, is reasonable because of the influence of noise in experiments and the different plates used for obtaining the reference data. In this experiment, we can find from Figure 8 and Figure 9 that the abnormal ratios are lower than 0.85 or higher than 1.1 for the ray paths passing through the damaged region. Therefore, it is acceptable to set $\alpha_{1}$ (or $\alpha_{3}$) and $\alpha_{2}$ (or $\alpha_{4}$) to be 0.85 and 1.1, respectively. We find that reasonably determined thresholds can increase the robustness of the technique. Moreover, it should be noted that the slightly different choices of the thresholds only affect the number and size of possible damaged regions in the first step; consequently, the number of unknowns and computational costs may also change. However, by performing the second step, basically, almost the same damage image can be reconstructed for the different reasonable thresholds.

**Figure 10:**
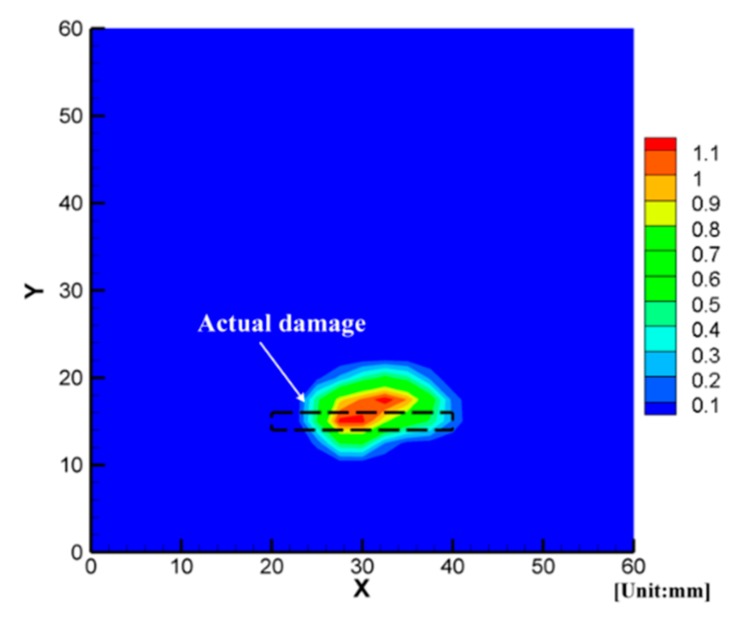
Image of an aluminum plate with a non-penetrating slit.

Based on the above thresholds, the number of unknowns in 24 × 24 = 576 grids decreased from 576 to 18, i.e., the attenuation change $\Delta x_{k}$ was only in the possible damaged area after the first step. Therefore, it could be easy to carry out the second step. Figure 10 illustrates the image result obtained from Equation (11) by using the least-square method. In Figure 10, the rectangular of the black dotted line represents the actual damage, and the value of the color bar denotes the change of attenuation coefficient $\Delta x_{k}$ in Equation (5). It can be considered that the area is damaged more seriously when the value of $\Delta x_{k}$ is larger. We can see that the location of the non-penetrating notch can be accurately detected, and the length of the notch can be estimated with about 15% error. However, it is a pity to see that the shape cannot be reconstructed. This error is mainly due to the larger grid size (2.5 mm) compared with the width of the notch (2 mm). However, reducing the grid size still cannot improve the result because the wavelength of excited A_0_ mode (15–20 mm) is much larger than the width of the notch. So, it can be predicted that the present technique cannot perfectly reconstruct the type of damage with a long and narrow shape.

### 4.2. A CFRP Laminated Plate with Impact-Induced Internal Delamination 

Delamination is one of the most common failure modes in composite laminates, and it may be formed because of various impact events, poor fabrication processes and fatigue. It is well-known that delamination may severely reduce the compressive strengths of structures made from laminated composite materials, so it is important to inspect it to ensure the safety of those structures.

**Figure 11:**
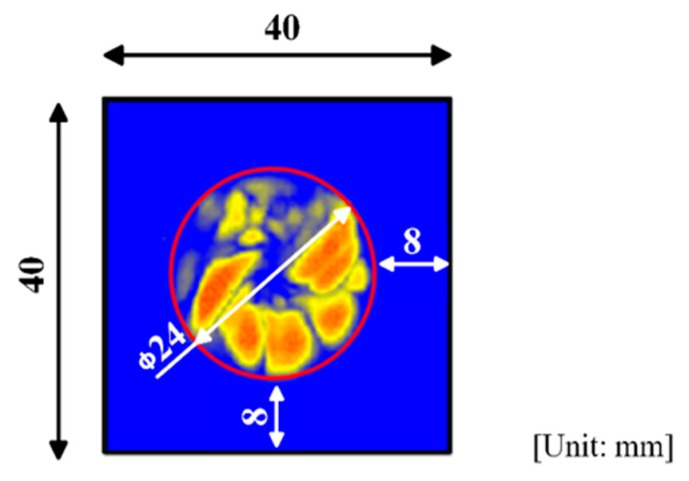
Image of ultrasonic C-scan inspection.

To validate the improved rapid reconstruction technique, a 32-layer quasi-isotropic CFRP laminated plate with a stacking sequence of [(45^0^/0^0^/–45^0^/90^0^)_4_]_s_ was used. The center of the plate was impacted by a rigid body of a lower semi-spherical shape. Its mass is 4.6 kg and the impact energy is 4.8 J. As a result, an internal delamination occurred. Firstly, a conventional ultrasonic C-scanning was performed by putting the specimen into a water bath with the scanning conditions: pitch = 0.5 mm and frequency ranging from 5 MHz to 10 MHz. From the ultrasonic C-scanning result as shown in Figure 11, it was found that there was an internal delamination in the CFRP laminated plate, and the diameter of the delamination was around 24.0 mm.

**Figure 12:**
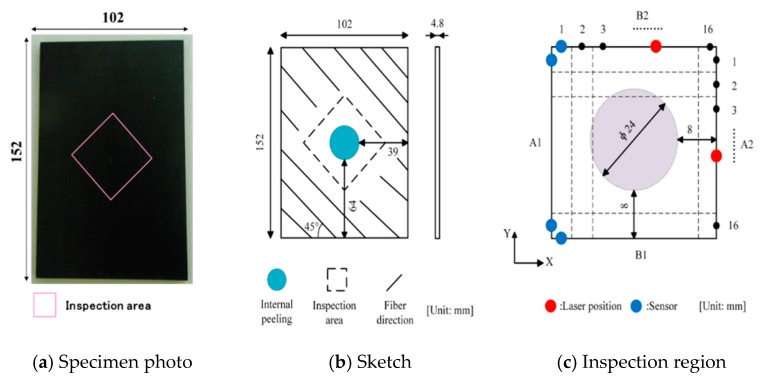
Model of CFRP laminated plate and inspection region.

The inspection regions of the CFRP laminated plate were determined by the internal delamination diameters identified by ultrasonic C-scanning inspection as shown in Figure 12. The detailed scanning scheme is shown in Figure 3a,b (*m* = 16, *n* = 16). The inspection region was determined as 40 × 40 ${\mathrm{mm}}^{2}$ and divided into 16 × 16 grids with a side length of 2.5 mm. As a result, only 16 × 4 = 64 waveform data were used in this inspection.

Since the wave attenuation strongly depends on the relationship between the wave propagation direction and the fiber direction of surface ply, it is necessary to consider the effect of the angle between the wave propagation direction and the fiber direction of surface ply on the reference data. Another intact quasi-isotropic CFRP laminated plate of the same stacking sequence as the specimen for the impact test was used to obtain the reference data on the basis of an experimental scheme in Figure 4. The inner radius *R*_1_ and the outer radius *R*_2_ of the ring were set to be 40 mm and 55 mm. There were two parameters needing to be recorded for each set of wave signals: the distance between the point of irradiation and the AE sensor, and the angle between the fiber direction of surface ply and the connecting line between the point of irradiation and the AE sensor. By extracting the amplitude from each set of wave signals, the reference data could be obtained by a bi-linear interpolation technique.

**Figure 13:**
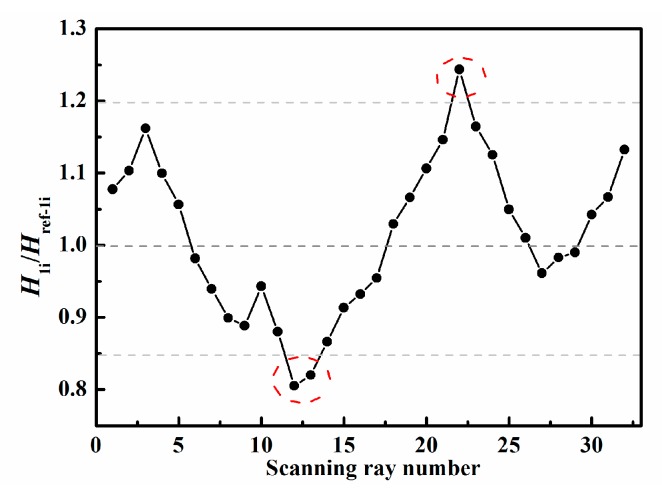
Ratio curve of the ray paths to the bottom-left corner for the front side.

**Figure 14:**
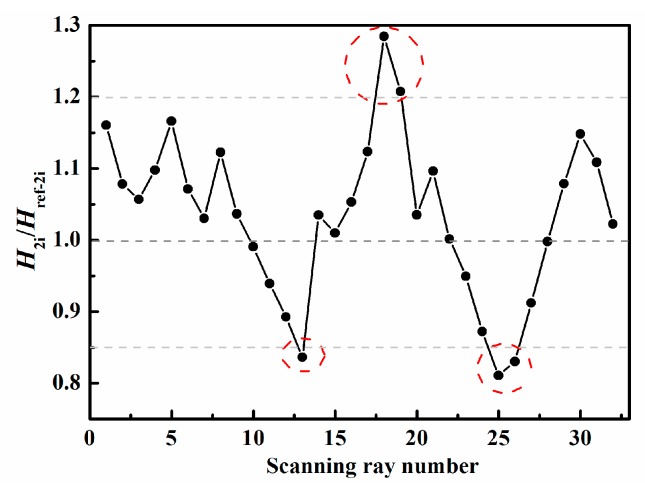
Ratio curve of the ray paths to the upper-left corner for the front side.

Therefore, the ratio of the measured value to the reference value could be calculated, and the thresholds could be determined according to the maximal and minimum values of the ratio values. The ratio curves of the ray paths to the bottom-left corner in Figure 3a and of the ray paths to the upper-left corner in Figure 3b are shown in Figure 13 and Figure 14, from which we could set $\alpha_{1}=\alpha_{3}=0.85$ and $\alpha_{2}=\alpha_{4}=1.2$. Then the possible damaged region could be obtained in the first step.

**Figure 15:**
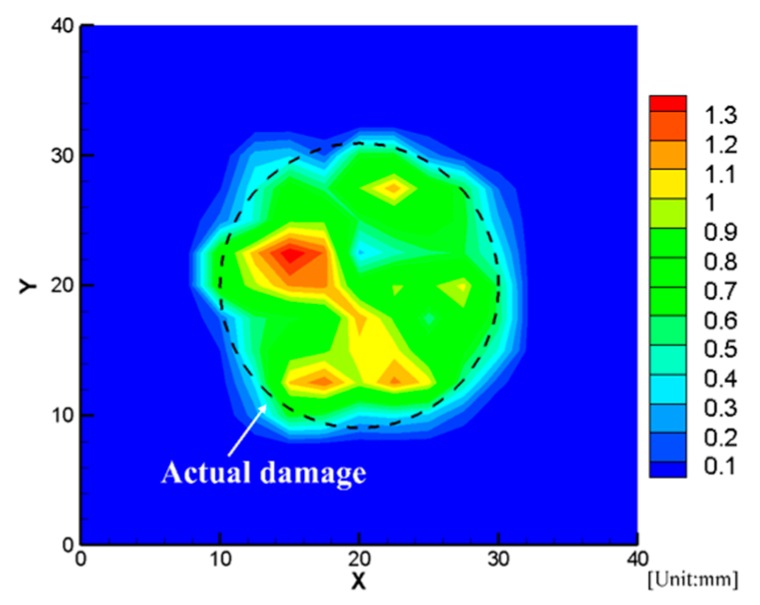
Reconstructed delamination images.

Next, the detailed damage images were reconstructed as stated in Section 3. Figure 15 presents the reconstructed delamination images with a comparison of ultrasonic C-scanning inspection results (black dotted lines). We can find that the delamination could be reconstructed successfully using the proposed rapid reconstruction technique. Finally, we can conclude that the present technique is effective for damage evaluation.

## 5. Conclusions

In this work, a pulse laser-combined LWT technique was proposed to identify damage, including damage size estimation for plate-like structures. A two-stage reconstruction algorithm which requires reference data was put forward. In stage one, the suspected damage area was identified by a comparison of the amplitude of the testing signal data and reference data. In stage-two, the damage image was reconstructed using an inverse approach based on the least-square method. Two experiments on an aluminum plate with a non-penetrating notch and a CFRP laminated plate with impact-induced delamination were carried out to validate the effectiveness of the proposed technique. The results showed that for the aluminum plate, only the location and length could be evaluated, but the shape of the notch could not be reconstructed due to the too small width of the notch compared with the wavelength of the excited waves. The result also implied that the proposed technique might not be suitable for the type of damage with a long and narrow shape. For the CFRP-laminated plate, the image of the internal delamination was reconstructed successfully, which shows the advantage of the proposed technique for anisotropic material.

## Figures and Tables

**Table 1:** Material properties of the aluminum plate.

Young’s Modulus *E* (Gpa)	Poisson’s Ratio *v*	*ρ* (kg/m^3^)
70	0.35	2700

**Table 2:** Material properties of the CFRP plate.

*E*_1_ (Gpa)	*E*_2_ (Gpa)	*v*_12_	*v*_23_	*G*_12_ (Gpa)	*ρ* (kg/m^3^)
135	10.5	0.25	0.4	5.5	1489

## References

[1] Tiwari K.A., Raisutis R., Samaitis V. (2017). Hybrid signal processing technique to improve the defect estimation in ultrasonic non-destructive testing of composite structures. Sensors.

[2] Tiwari K.A., Raisutis R. (2018). Identification and characterization of defects in glass fiber reinforced plastic by refining the guided Lamb waves. Materials.

[3] Zeng L., Huang L., Lin J. (2019). Damage imaging of composite structures using multipath scattering Lamb waves. Compos. Struct..

[4] Eremin A., Golub M., Glushkov E., Glushkova N. (2019). Identification of delamination based on the Lamb wave scattering resonance frequencies. NDT E Int..

[5] Grangeat P. (2013). Tomography.

[6] Nathan B., Vladimir Y. (2018). Electromagnetic and Acoustic Wave Tomography: Direct and Inverse Problems in Practical Applications.

[7] Jansen D.P., Hutchins D.A. Lamb wave tomography. Proceedings of the IEEE Ultrasonics Symposium.

[8] Su Z., Lin Y. (2009). Identification of Damage using Lamb Waves: From Fundamentals to Applications.

[9] Hutchins D., Jansen D., Edwards C. (1993). Lamb-wave tomography using non-contact transduction. Ultrasonics.

[10] Jansen D., Hutchins D., Mottram J. (1994). Lamb wave tomography of advanced composite laminates containing damage. Ultrasonics.

[11] McKeon J.C.P., Hinders M.K. (1999). Parallel projection and crosshole Lamb wave contact scanning tomography. J. Acoust. Soc. Am..

[12] Malyarenko E.V., Hinders M.K. (2000). Fan beam and double crosshole Lamb wave tomography for mapping flaws in aging aircraft structures. J. Acoust. Soc. Am..

[13] Leonard K.R., Malyarenko E.V., Hinders M.K. (2002). Ultrasonic Lamb wave tomography. Inverse Probl..

[14] Hou J., Leonard K.R., Hinders M.K. (2004). Automatic multi-mode Lamb wave arrival time extraction for improved tomographic reconstruction. Inverse Probl..

[15] Leonard K.R., Hinders M.K. (2005). Lamb wave tomography of pipe-like structures. Ultrasonics.

[16] Huang S., Zhang Y., Wang S., Zhao W., Jiang X. (2016). Multi-mode electromagnetic ultrasonic Lamb wave tomography imaging for variable-depth defects in metal plates. Sensors.

[17] Chen L., Xiao Q., Liang W., Hong J., Zou X. (2018). A time-of-flight revising approach to improve the image quality of Lamb wave tomography for the detection of defects in composite panels. Sci. Eng. Compos. Mater..

[18] Seher M., Huthwaite P., Lowe M.J.S. (2016). Experimental studies of the inspection of areas with restricted access using A0 Lamb wave tomography. IEEE Trans. Ultrason. Ferroelectr. Freq..

[19] Zhao J., Miao X., Li F., Li H. (2018). Probabilistic diagnostic algorithm-based damage detection for plates with non-uniform sections using the improved weight function. J. Vib. Eng. Technol..

[20] Malyarenko E.V., Hinders M.K. (2001). Ultrasonic Lamb wave diffraction tomography. Ultrasonics.

[21] Belanger P., Cawley P., Simonetti F. (2010). Guided wave diffraction tomography within the born approximation. IEEE Trans. Ultrason. Ferroelectr. Freq..

[22] Ng C.-T. (2015). A two-stage approach for quantitative damage imaging in metallic plates using Lamb waves. Earthq. Struct..

[23] Chan E., Rose L.R.F., Wang C.H. (2015). An extended diffraction tomography method for quantifying structural damage using numerical Green’s functions. Ultrasonics.

[24] Ren Y., Qiu L., Yuan S., Su Z.Q. (2017). A diagnostic imaging approach for online characterization of multi-impact in aircraft composite structures based on a scanning spatial-wavenumber filter of guided wave. Mech. Syst. Signal Process..

[25] Tant K.M.M., Galetti E., Mulholland A.J., Curtis A., Gachagan A. (2018). A transdimensional Bayesian approach to ultrasonic travel-time tomography for non-destructive testing. Inverse Probl..

[26] Qiu L., Yuan S., Zhang X., Wang Y. (2011). A time reversal focusing based impact imaging method and its evaluation on complex composite structures. Smart Mater. Struct..

[27] Morii M., Hu N., Fukunaga H., Li J.H., Liu Y.L., Atobe S., Alamusi, Qiu J.H. (2011). A new inverse algorithm for tomographic reconstruction of damage images using Lamb waves. Comput. Mater. Con..

[28] Hu B., Hu N., Li L., Li W., Tang S., Li Y., Peng X., Homma A., Liu Y., Wu L. (2014). Tomographic reconstruction of damage images in hollow cylinders using Lamb waves. Ultrasonics.

[29] Zhou C., Su Z.Q., Cheng L. (2011). Probability-based diagnostic imaging using hybrid features extracted from ultrasonic Lamb wave signals. Smart Mater. Struct..

[30] Stark H., Woods J., Paul I., Hingorani R. (1981). Direct Fourier reconstruction in computer tomography. IEEE Trans. Acoust. Speech Signal Process..

[31] Zhuang T., Leng S., Nett E.B. (2004). Fan-beam and cone-beam image reconstruction via filtering the back-projection image of differentiated projection data. Phys. Med. Boil..

[32] Cebeiro J., Morvidone M., Nguyen M.K. (2016). Back-projection inversion of a conical radon transform. Inverse Probl. Sci. Eng..

[33] Gordon R., Bender R., Herman G.T. (1970). Algebraic Reconstruction Techniques (ART) for three-dimensional electron microscopy and X-ray photography. J. Theor. Biol..

[34] Gilbert P. (1972). Iterative methods for the three-dimensional reconstruction of an object from projections. J. Theor. Biol..

[35] Rosalie C., Chan A., Chiu W.K., Galea S.C., Rose F., Rajic N. (2005). Structural health monitoring of composite structures using stress wave methods. Compos. Struct..

[36] Kazantsev D., Pickalov V. (2018). New iterative reconstruction methods for fan-beam tomography. Inverse Probl. Sci. Eng..

[37] Zhao Z., Chang F., Li B. (2016). Ultrasonic CT reconstruction based on improved maximum likelihood expectation maximization algorithm. J. Electron. Meas. Instrum..

[38] Zhang F., Zhang Q., Cui X., Dong C., Liu Y., Liu Y., Sun W., Gui Z. (2014). Low-dose computed tomography reconstruction algorithm based on patch similarity and maximum likelihood expectation maximization. J. Comput. Appl..

